# Human Wharton’s jelly mesenchymal stem cells promote skin wound healing through paracrine signaling

**DOI:** 10.1186/scrt417

**Published:** 2014-02-24

**Authors:** Anna I Arno, Saeid Amini-Nik, Patrick H Blit, Mohammed Al-Shehab, Cassandra Belo, Elaine Herer, Col Homer Tien, Marc G Jeschke

**Affiliations:** 1 Plastic Surgery Department and Burn Unit, Vall d’Hebron University Hospital, Universitat Autònoma de Barcelona, Passeig de la Vall d'Hebron 119-129, 08035 Barcelona, Spain; 2 Ross Tilley Burn Centre and Sunnybrook Research Institute, Sunnybrook Health Sciences Centre, University of Toronto, 2075 Bayview Avenue, Toronto, ON M4N 3M5, Canada; 3 Gynecology and Obstetrics Department, Sunnybrook Health Sciences Centre, University of Toronto, 2075 Bayview Avenue, Toronto, ON M4N 3M5, Canada; 4 Canadian Forces Health Services; Trauma, Emergency and Critical Care Program, Sunnybrook Health Sciences Centre, University of Toronto, 2075 Bayview Avenue, Toronto, ON M4N 3M5, Canada

## Abstract

**Introduction:**

The prevalence of nonhealing wounds is predicted to increase due to the growing aging population. Despite the use of novel skin substitutes and wound dressings, poorly vascularized wound niches impair wound repair. Mesenchymal stem cells (MSCs) have been reported to provide paracrine signals to promote wound healing, but the effect of human Wharton’s jelly-derived MSCs (WJ-MSCs) has not yet been described in human normal skin.

The aim of this study is to examine the effects of human WJ-MSC paracrine signaling on normal skin fibroblasts *in vitro*, and in an *in* v*ivo* preclinical model.

**Methods:**

Human WJ-MSCs and normal skin fibroblasts were isolated from donated umbilical cords and normal adult human skin. Fibroblasts were treated with WJ-MSC-conditioned medium (WJ-MSC-CM) or nonconditioned medium.

**Results:**

Expression of genes involved in re-epithelialization (*transforming growth factor-β2*), neovascularization (*hypoxia-inducible factor-1α*) and fibroproliferation (*plasminogen activator inhibitor-1*) was upregulated in WJ-MSC-CM-treated fibroblasts (*P* ≤ 0.05). WJ-MSC-CM enhanced normal skin fibroblast proliferation (*P* ≤ 0.001) and migration (*P* ≤ 0.05), and promoted wound healing in an excisional full-thickness skin murine model.

**Conclusions:**

Under our experimental conditions, WJ-MSCs enhanced skin wound healing in an *in vivo* mouse model.

## Introduction

Nonhealing or chronic wounds represent an increasingly prevalent and costly public health issue. As a consequence of longer lifespans due to improvements in acute care, the number of patients who suffer diabetes and other chronic aging-related diseases is growing considerably. There is a link with aging-associated diseases and wound healing impairment [1]. On the other hand, other wound healing challenges also arise at any age because of accidents, burns and other traumatic injuries.

Mesenchymal stem cells (MSCs) appear to emerge as a promising wound healing therapy. Paracrine signaling, such as the release of factors that promote angiogenesis, immunomodulation and recruitment of endogenous tissue stem/progenitor cells, as well as differentiation, have been described as possible mechanisms underlying the positive wound healing effects of MSCs [2,3]. From the different available sources of MSCs, the umbilical cord represents a cost-effective, productive, feasible, accepted, and universal source to isolate MSCs, and is considered advantageous compared with bone marrow-derived mesenchymal stem cells (BM-MSCs) and adipose-derived MSCs for some researchers [4]. Previous studies with umbilical cord Wharton’s jelly-derived mesenchymal stem cells (WJ-MSCs) have demonstrated that they represent a high-yield source of young, nontumorigenic and immunomodulatory cells that may be allotransplanted to regenerate liver, heart, bone, cartilage, fat, pancreas, neural, vascular/endothelial and skin components [5]. WJ-MSCs isolated from goats have been demonstrated to accelerate wound closure in animals from the same species, while minimizing granulation tissue and inflammation [6]. Human WJ-MSCs decrease lung [7], kidney [8] and liver [9] fibrosis, and have been shown to be able to differentiate into sweat gland-like cells and may therefore promote skin regeneration [10]. WJ-MSCs secrete proangiogenic and wound healing promoting factors, such as transforming growth factor beta (TGF-β), vascular endothelial growth factor (VEGF), platelet-derived growth factor, insulin-like growth factor-I, interleukin (IL)-6 and IL-8, among others [11]. Paracrine effects appear to be responsible for the wound healing promoting effects of WJ-MSCs, at least in mice [12]. However, to date, there are no reports regarding the use of human WJ-MSCs in human skin wounds.

The novelty of this study lies in the use of a promising stem cell type, the WJ-MSC, which has yet not been studied in the context of human skin, and investigating the *in vitro* effect on human skin fibroblasts, as a means to develop a new therapeutic strategy to aid in wound healing. Specifically, the aim of this study is to analyze the wound healing effects of human WJ-MSC paracrine signaling on human normal skin fibroblasts *in vitro*, and to examine the application of WJ-MSC conditioned medium (CM) into an *in vivo* mouse wound healing model.

## Materials and methods

### Tissue sources

Normal skin was obtained from healthy donors undergoing plastic surgery procedures (mostly dermolipectomies), both men and women, excluding pregnant females. Umbilical cords were obtained from pregnant women after planned delivery through caesarian section. Tissues were obtained at the Department of Plastic Surgery and the Department of Obstetrics and Gynecology, respectively, at Sunnybrook Health Sciences Centre, University of Toronto, Toronto, Ontario, Canada. Tissue specimens were collected following the Declaration of Helsinki Principles, following Toronto Academic Health Sciences Network (TAHSN) and University of Toronto-affiliated Sunnybrook Research Institute and Sunnybrook Health Sciences Centre Institutional Ethics Review Board approval, and after getting patient signed informed consent.

### Cell culture

Primary human normal skin fibroblasts and human WJ-MSCs were obtained from skin tissue samples and umbilical cords, respectively. Skin was dissected to remove any underlying fat, cut into small explant pieces of 2 to 4 mm, and cultured in small dishes. WJ-MSCs were isolated from umbilical cords by gentle dissection of previous sectioned small cord pieces, discarding the outer or epithelial layer, according to earlier described methods [13]. Explants and MSCs were further subcultured in Petri dishes at a density of 3,200 cells/cm^2^ for 7 days before further passaging. When fibroblasts and/or WJ-MSCs reached 70% confluence, usually within 1 week, they were trypsinized with 0.05% trypsin/0.025% ethylenediamine tetraacetic acid v/v in preparation for subculture. Fibroblasts were subcultured in 75 cm^2^ tissue culture flasks at a density of 4,500 cells/cm^2^. Tissue culture plasticware were purchased from BD Falcon™ (Bedford, MA, USA), and all tissue culture media and supplements were obtained from Wisent Inc. (St-Jean-Baptiste, QC, Canada), unless otherwise stated. Fibroblast culture medium consisted of high-glucose Dulbecco’s modified Eagle’s medium (DMEM) supplemented with 10% fetal bovine serum (FBS) and 1% antibiotic–antimycotic solution. WJ-MSC culture medium consisted of CMRL (Gibco, Carlsbad, CA, USA) with 10% FBS, 2% antibiotic–antimycotic solution and 1% l-glutamine. Media were changed every 48 hours. Collected tissues and cells were cultured at 37°C in a humidified atmosphere with 5% carbon dioxide.

### Characterization of human Wharton's jelly-derived MSCs

The cells isolated from the Wharton’s jelly of the umbilical cord were examined to confirm their MSC characteristics. Flow cytometry for MSC cell surface markers (CD90^+^, CD73^+^, CD105^+^, CD45^–^, CD14^–^, CD34^–^, CD19^–^ and HLA-DR^–^) was performed (Additional file 1). Cells were differentiated into the three main mesenchymal lineages – adipogenic, osteogenic and chondrogenic (Additional file 1). For adipogenic differentiation, cells were seeded at a density of 3,000 cells/cm^2^ in 24-well plates (BD) with low-glucose DMEM (Wisent Inc., St-Jean-Baptiste, QC, Canada) medium supplemented with 10% FBS, 1% antibiotic–antimycotic solution, 1 mM 3-isobutyl-1-methylxanthine (Sigma-Aldrich, Saint Louis, MO, USA), 10 μg/ml insulin (SAFC, Saint Louis, MO, USA), 60 μM indomethacin (Sigma-Aldrich), and 1 μM dexamethasone (Sigma-Aldrich). Cultures of cells in low-glucose DMEM medium supplemented with 10% FBS served as a negative control. Lipid accumulation was identified by oil red O staining: 0.3 g oil red O (Sigma-Aldrich) dissolved in 100 ml isopropanol (Sigma-Aldrich), diluted to 60% with distilled water.

For osteogenic differentiation, cells were also seeded at a density of 3,000 cells/cm^2^ in 24-well plates with low-glucose DMEM supplemented with 10% FBS, 1% antibiotic–antimycotic solution, 0.05 mM ascorbic acid-2-phosphate (Wako Pure Chemicals Industry Ltd, Osaka, Japan), 10 mM β-glycerophosphate (Sigma-Aldrich), and 100 nM dexamethasone (Sigma-Aldrich). Alizarin red staining (Sigma-Aldrich) was used to identify osteogenic cells (2 g alizarin red dissolved in 100 ml distilled water). For chondrogenic differentiation, cells were seeded in 15 ml polypropylene tubes (2 × 10^5^ cells per tube; BD Falcon, Bedford, MA, USA) with low-glucose DMEM supplemented with 10% FBS, 1% antibiotic–antimycotic solution, 1 mM sodium pyruvate (Sigma-Aldrich), 0.1 mM ascorbic acid-2-phosphate (Wako Pure Chemicals Industry Ltd), 1% insulin–transferrin–selenium (Cellgro, Manassas, VA, USA), 100 nM dexamethasone (Sigma-Aldrich), and 10 ng/ml TGF-β3 (Shenandoah Biotechnology, Inc., Warwick, PA, USA). Chondrocyte pellets were identified with Safranin O staining: 0.1 g Safranin O (Sigma-Aldrich), dissolved in 100 ml distilled water.

### Human normal fibroblasts and Wharton's jelly-derived MSC one-way paracrine signaling indirect co-culture

First, primary human fibroblasts were seeded into six-well plates (Grenier-Bio-One Cellstar, Frieckenhausen, Germany) at a density of 22,000 cells/cm^2^ with DMEM. To design a one-way indirect or paracrine co-culture system between human normal skin fibroblasts and WJ-MSCs, the WJ-MSCs were separately seeded at the same cell density in the upper three wells of a six-well plate with CMRL media; the lower three wells of the same six-well plate were filled with CMRL medium alone. When refreshing the media (once every other day), the six-well plate containing the fibroblasts was filled with the medium from the WJ-MSC six-well plate: the three upper wells (treatment wells) with the WJ-MSC-CM, and the three lower wells (control wells) with the CMRL media alone. On day 5 of culture, the amount of FBS in the medium was reduced from 10 to 2%, to avoid TGF-β1 false measurements. On day 7, total RNA extraction was started for further gene expression studies. Experiments were performed with low-passage cells (less than passage 5) and in triplicate (unless otherwise stated), on one set of cells from different patients.

### RNA isolation and real-time quantitative polymerase chain reaction

For RNA isolation, cells were lysed using TRIzol reagent (Invitrogen, Carlsbad, CA, USA), and the RNeasy MicroKit was used (Qiagen, Inc., Valencia, CA, USA) according to the manufacturer’s instructions. The total RNA yield was determined using a NanoDrop-2000 spectrophotometer (ThermoScientific, Waltham, MA, USA). cDNA was synthesized in a thermocycler (AB Applied Biosystems, Foster City, CA, USA), after mixing 10 μg RNA and a master mix prepared with the high-capacity cDNA synthesis reverse transcription kit (AB Applied Biosystems). Real-time polymerase chain reaction (PCR) was conducted using SYBR® Green PCR Master Mix (Applied Biosystems) to relatively quantify the mRNA transcript products of the following genes of interest: *TGF-β1*, *TGF-β2*, *TGF-β3*, *plasminogen activator inhibitor-1*, *connective tissue growth factor*, *fibroblast growth factor-2*, *hypoxia-inducible factor-1α*, *VEGF*, *collagen I*, *collagen III* and *decorin*. 18S was used as housekeeping gene. The primer sequences of the above genes used are listed in Additional file 2. Amplification and analysis of cDNA fragments were carried out using the StepOnePlus RT-PCR System (AB Applied Biosystems).

Relative gene expression was measured as the cycle threshold and was normalized with individual housekeeping gene control cycle threshold values. Quantitative PCR was loaded in duplicate, and cycle threshold values from triplicates of the same treatment group sample were averaged. The ΔΔCt method was used to report quantitative PCR results.

### Proliferation assay: Ki67 antigen staining

Normal skin fibroblasts and WJ-MSCs were seeded in different eight-chamber culture slides at a cell density of 715 cells/cm^2^ and cultured for 7 days, following methods described above. Briefly, the media from the wells containing WJ-MSC (WJ-MSC-CM) were used to fill the wells from the normal skin fibroblasts from the treated group every other day, whereas wells of the control group were filled with WJ-MSC nonconditioned media.

#### Immunofluorescence

Cells were washed with phosphate-buffered saline (PBS) and fixed for 15 minutes in 4% paraformaldehyde (Alfa Aesar, Karlsruhe, Germany). Fixed cells were washed in PBS and permeabilized for 10 minutes with PBS/0.5% Triton X-100 solution. After another washing step, cells were blocked for 30 minutes with 1% bovine serum albumin in PBS/0.5% Triton X-100. A monoclonal mouse anti-human Ki67 (1:100, clone MIB-1; Dako, Markham, ON, Canada) primary antibody was added and incubated overnight at 4°C. After washing with PBS, the secondary antibody was added in 1% bovine serum albumin in PBS/0.5% Triton X-100 and incubated for 1 hour at room temperature in the dark (Alexa Fluor 488 donkey anti-mouse, 1:500; Life Technologies, Eugene, OR, USA). After three final washes with PBS, slides were mounted with Vectashield mounting medium with 4′,6-diamidino-2-phenylindole (DAPI; Vector Laboratories, Burlingame, CA, USA). Cells were examined and photographed using an Apotome Axiovert fluorescent imaging system at 10× magnification (Zeiss, Oberkochen, Germany). Three images were taken per well and two wells were imaged per treatment. Quantification was performed by counting the number of Ki67-positive cells in the high-power field as well as the total number of DAPI-positive nuclei. Data are presented as means with 95% confidence intervals of duplicate measurements for three different normal skin samples.

### Terminal transferase TdT-mediated dUTP biotin end-labeling apoptosis assay

Normal fibroblasts cultured with WJ-MSC-CM and with nonconditioned medium were seeded into eight-chamber culture slides at a cell density of 3,000 cells/cm^2^ for half a week. The terminal transferase TdT-mediated dUTP biotin end-labeling apoptosis kit (Promega, Fitchburg, WI, USA) was used as per the manufacturer’s instructions.

Briefly, cells were fixed in 4% paraformaldehyde for 25 minutes at 4°C and washed with PBS. They were permeabilized with 0.25% Triton X-100 in PBS for 5 minutes, and then washed with PBS. Cells were incubated with equilibration buffer for 10 minutes, followed by labeling with terminal deoxynucleotidyl transferase reaction mix (10% nucleotide mix, 0.02% rTdT enzyme in equilibration buffer) for 1 hour at 37°C in humidified chamber. Cells were immersed in 2× SSC for 15 minutes to stop the reaction, followed by washes with PBS. Slides were mounted with Vectashield mounting medium with DAPI (H-1200; Vector Laboratories). Images were taken on an Apotome Axiovert fluorescent imaging system at 10× magnification (Zeiss); three images were taken per well. Quantification was performed by counting the number of terminal transferase TdT-mediated dUTP biotin end-labeling-positive and DAPI-positive nuclei, or apoptotic and alive cells, respectively.

### Cell migration study: scratch wound assay

Normal skin fibroblasts were seeded in four-chamber culture slides at a cell density of 1,000 cells/cm^2^, with WJ-MSC-CM (or non-conditioned medium as control) for 48 hours. Two scratches were performed with a 200 μl pipette tip. After 24 hours, cells were fixed with 4% paraformaldehyde. The staining protocol followed the same procedure as the aforementioned proliferation assay. Briefly, cells were incubated with phalloidin antibody conjugated to fluorescein isothiocyanate (1:30; Invitrogen, Eugene, OR, USA) in blocking solution for 1 hour. Cells were washed three times with PBS and mounted with Vectashield mounting medium with DAPI. Images were taken on laser scanning META 510 confocal microscope (Zeiss) with 5× magnification. Three images were taken per scratch. Quantification was performed using ImageJ software (National Institutes of Health, Bethesda, MD, USA). A set area with a height of 0.5 mm and a width spanning the high-power field was placed in the center of the scratch, and the cells within this area were counted as the cells in scratch zone.

### *In vivo* wound healing model

Eight BALB/c mice (13 weeks old, male, body weight 28 to 34 g) were obtained from Jackson Laboratory under the guidelines of the Sunnybrook Research Institute and Sunnybrook Health Sciences Animal Policy and Welfare Committee of the University of Toronto. Animal procedures were reviewed and approved by Sunnybrook Research Institute and Sunnybrook Health Sciences Centre at University of Toronto animal care and use committee. Animals were anesthetized and back cutaneous hair was removed by electrical shaving under anesthesia as stated in the Animal Protocol. Two pairs of 4 mm diameter full-thickness skin excisional wounds were created on each side of the midline. The animals were randomly divided into two groups: treatment (WJ-MSC-CM and Matrigel; BD Biosciences, San Jose, CA, USA) and sham (nonconditioned medium and Matrigel). Matrigel was of high concentration and was applied dropwise in liquid form and then allowed to gel. Each wound topically received 100 μl treatment or sham mix.

#### Wound analysis

Wound measurements were taken and wound closure was examined in a timely manner on days 1, 3 and 7. Wounds with a complete re-epithelialization were considered healed wounds.

Mice were sacrificed at day 7, when skin biopsies including the wound/scar and 2 mm of satellite skin were harvested for further histologic analysis. Twenty-four hours before sacking, animals received an intraperitoneal injection of bromodeoxyuridine (BrdU) (Calbiochem, San Diego, CA, USA).

#### Histologic examination

Tissue specimens were fixed in 10% buffered formalin overnight at room temperature, preserved in 70% ethanol and embedded in paraffin. Specimens were cut into 5 μm sections. Tissue specimens were cut simultaneously at different sites, the center or midline and both sides, eliciting a cross-section through the whole wound and satellite area. A serial section of the scar or healing wound was performed. The largest wound diameter or central wound section was stained for trichrome staining. Trichrome reagents were from EMS (Hatfield, PA, USA) unless otherwise stated. Briefly, paraffin-embedded slides were deparaffinized with citrosol, followed by rehydration through 100%, 95%, 70% and 50% ethanol to water. Slides were placed in Bouin’s solution (26367–01; EMS, Hatfield, PA, USA) overnight at room temperature and washed next. Hematoxylin stain (HHS16; Sigma, Saint Louis, MO, USA) and Biebrich scarlet-acid fuchsin solution were applied sequentially for 10 minutes. Washes were performed after each stain addition. Slides were differentiated in phosphomolybdic–tungstic acid for 15 minutes, and were transferred to aniline blue for 5 minutes. They were next rinsed and differentiated in 1% acetic acid for 2 minutes. Slides were dehydrated through 95% ethanol and absolute ethanol followed by clearing in citrosol. Slides were mounted with SHUR/Mount xylene-based liquid mounting media (Triangle Biomedical Sciences, Durham, NC, USA). Images were acquired using a Zeiss Axiovert 200 light microscope at 10× and 40× magnification. Quantification was carried out using merged 10× images to measure the wound bed and satellite area.

For immunohistochemistry staining, paraffin-embedded skin tissue slides were deparaffinized with xylene followed by rehydration. Antigen decloaker (1×; Biocare Medical, Concord, CA, USA) was added to the slides in a preheated decloaking chamber for 4 minutes at 110°C. For BrdU staining, samples were denatured with 1.5 N HCl for 30 minutes at 37°C and neutralized with 0.1 M borate buffered twice for 5 minutes. Samples were blocked with 3% H_2_O_2_ for 10 minutes, and then washed with washing buffer (0.05 M Tris–HCl, 0.15 M NaCl, 0.05% Tween 20 in deionized water). The primary antibody (mouse monoclonal anti-BrdU, 1:200; Cell Signaling, Beverly, MA, USA) was diluted in PBS and incubated at room temperature for 1 hour. Slides were then incubated for 15 minutes first with MACH3 mouse probe (Biocare Medical), and secondly with MACH3 rabbit or mouse horseradish peroxidase polymer, with before and after washes. The betazoid diaminobenzidine chromogen kit (Biocare Medical) was mixed and added for 5 minutes or until brown stain was noticeable. The reaction was terminated with running water. Nuclear staining was carried out with hematoxylin for 30 seconds, followed by differentiation with three dips in 1.5% acid alcohol and bluing in 0.1% sodium bicarbonate for 10 seconds. Sections were dehydrated through 95% and absolute ethanol to citrosol and mounted with SHUR/Mount as described previously. Images were acquired using a Zeiss Axiovert 200 light microscope at 10× magnification to image the whole section followed by 40× magnification to further focus on the wound margins and the wound center. The higher magnification images of BrdU staining were quantified by counting using ImageJ software, and normalized to the number of cells in the high-power field.

### Statistical analysis

The statistical comparisons between the groups were performed using an unpaired Student’s *t* test with GraphPad Prism software (GraphPad, LaJolla, CA, USA). Two-tailed *P* ≤ 0.05 was considered significant. Data were graphically expressed as the mean of the target group ± the standard error of the mean or 95% confidence interval.

## Results

### Wharton's jelly-derived MSCs enhance the expression of wound healing genes by paracrine signaling

**Figure 1:**
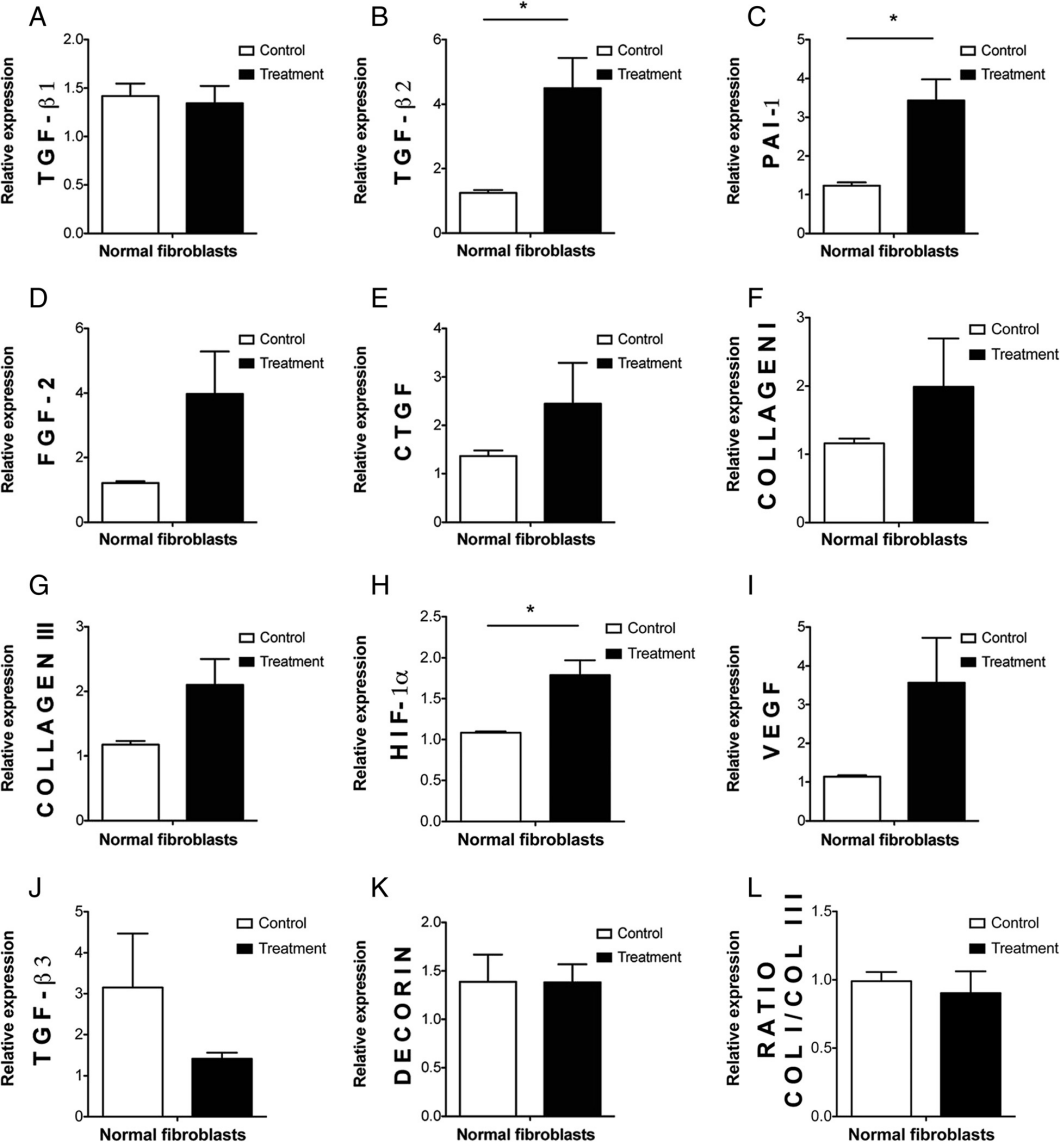
**Upregulation of wound healing genes by human Wharton's jelly-derived mesenchymal stem cell conditioned medium in human normal skin fibroblasts. (A-L)** mRNA transcript expression relative to 18S after 7 days of culture of human normal skin fibroblasts with Wharton's jelly-derived mesenchymal stem cell conditioned medium (WJ-MSC-CM; treatment group) or nonconditioned medium (control group) from five different patients (but four patients for FGF-2 and three patients for collagen I, collagen III and decorin). Overall, WJ-MSC-CM enhanced a wound healing promoting phenotype in human normal skin fibroblasts in our culture conditions. *P ≤ 0.05. COL, collagen; CTGF, connective tissue growth factor; FGF-2, fibroblast growth factor-2; HIF-1α, hypoxia-inducible factor-1α; PAI-1, plasminogen activator inhibitor-1; TGF-β, transforming growth factor beta; VEGF, vascular endothelial growth factor*.*

Human WJ-MSCs upregulated the mRNA transcript expression of *TGF-β2*, *hypoxia-inducible factor-1α*, and *plasminogen activator inhibitor-1* genes (*P* ≤ 0.05) in normal skin fibroblasts in our culture conditions. Other genes involved in re-epithelialization, neovascularization and/or remodeling – including *VEGF*, *fibroblast growth factor-2*, *connective tissue growth factor*, *collagen I* and *collagen III* – were not changed. *Decorin* and *TGF-*β3 also remained unaffected (Figure 1).

### Normal skin fibroblasts proliferate faster when treated with Wharton's jelly-derived MSC conditioned medium

**Figure 2:**
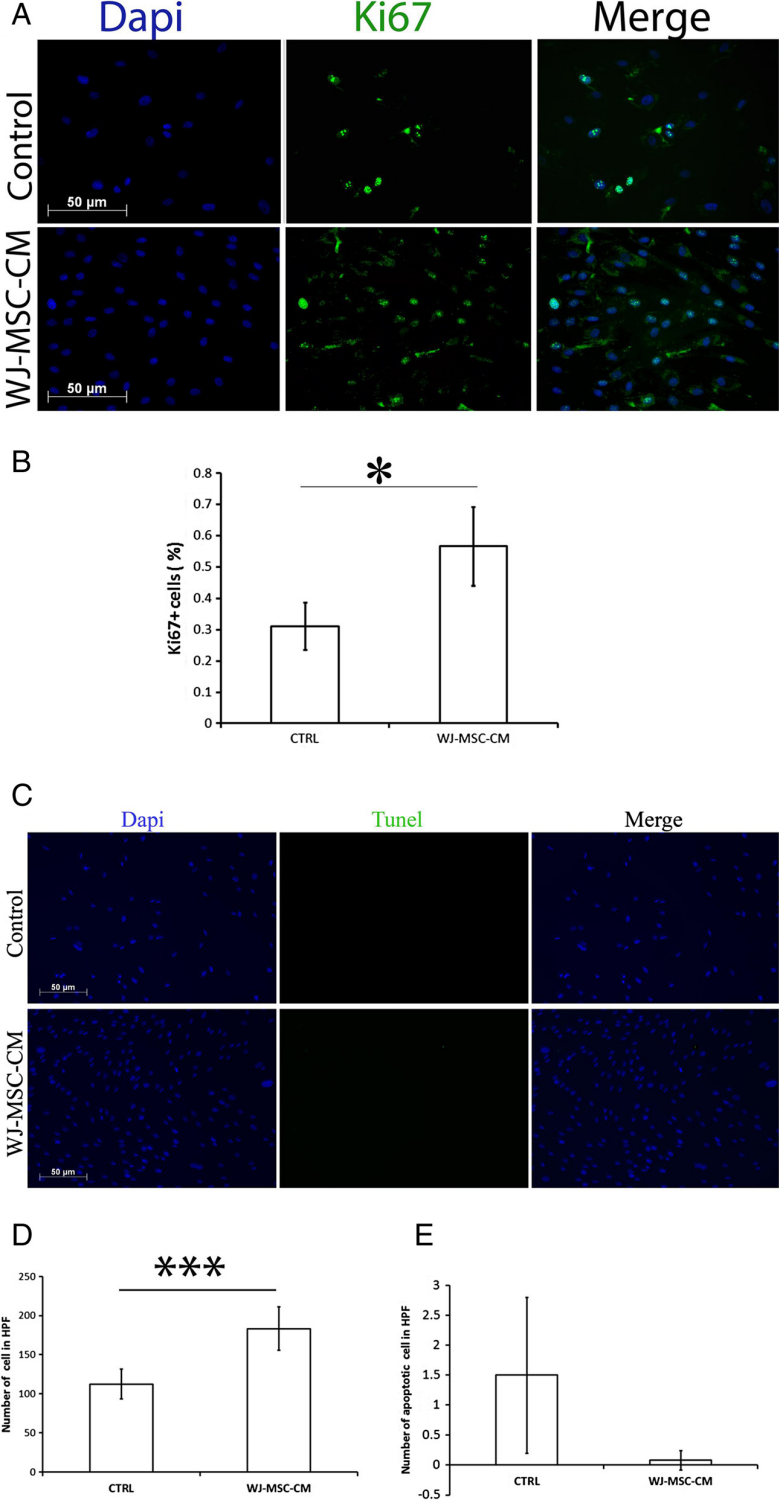
**Wharton's jelly-derived mesenchymal stem cell conditioned medium increases normal skin fibroblast proliferation, but does not affect apoptosis.** Cell proliferation was examined using Ki67 staining. **(A)** Wharton's jelly-derived mesenchymal stem cell conditioned medium (WJ-MSC-CM)-treated normal fibroblasts showed enhanced proliferative rates compared with the control group (quantified in **(B)**). **(C)** Terminal transferase TdT-mediated dUTP biotin end-labeling (TUNEL) staining of WJ-MSC-CM-treated human normal skin fibroblasts versus control (non-WJ-MSC-CM-treated) normal skin fibroblasts showed no significant difference in induction of apoptosis (quantified in **(E)**). Note that the total number of viable cells was significantly higher in the WJ-MSC-CM-treated cells compared with the non-WJ-MSC-CM-treated cells **(D)**. *n* = 3 samples per group. *P ≤ 0.05, ***P ≤ 0.001. DAPI, 4′,6-diamidino-2-phenylindole.

In our culture conditions, WJ-MSC-CM accelerated normal skin fibroblasts proliferation (*P* ≤ 0.001), as measured by a Ki67 proliferation assay (Figure 2A,B,C).

### Wharton's jelly-derived MSC conditioned medium does not induce apoptosis in normal skin fibroblasts

We did not find any significant modulation in the number of apoptotic fibroblasts treated with WJ-MSC-CM using a terminal transferase TdT-mediated dUTP biotin end-labeling assay, suggesting that WJ-MSC-CM does not appear to affect normal skin fibroblast apoptosis under our culture conditions (Figure 2D). An increased apoptosis rate could imply a delayed wound healing process, whereas a much decreased apoptosis rate could imply enhanced risk of keloid scars.

### Wharton's jelly-derived MSC conditioned medium promotes normal skin fibroblast migration and wound closure

To further examine whether the enhanced proliferation of fibroblasts treated with WJ-MSC-CM may also promote wound closure, a wound scratch assay was performed.

**Figure 3:**
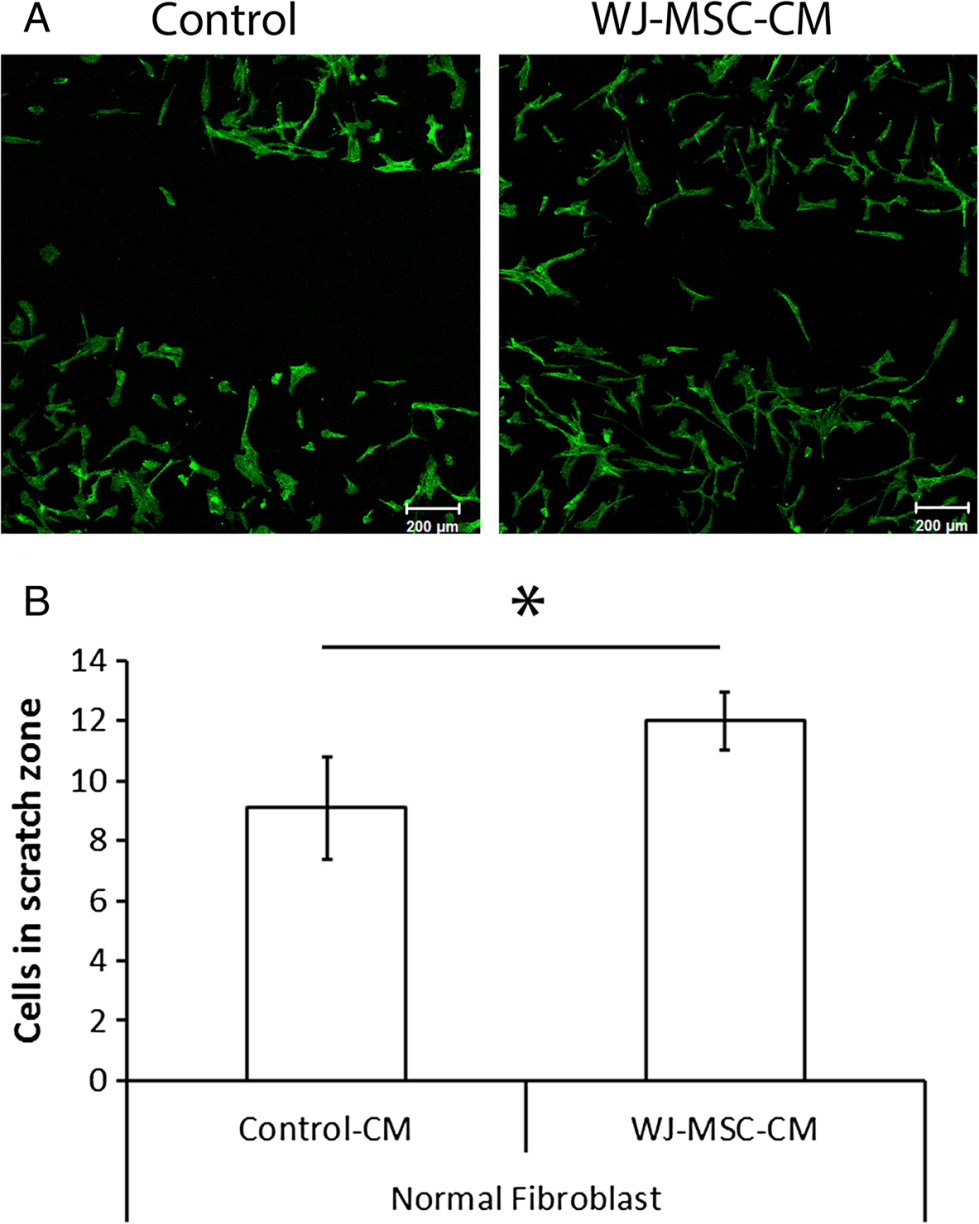
**Wharton's jelly-derived mesenchymal stem cell conditioned medium accelerates wound closure *in vitro*. (A)**, **(B)** Scratch wound assay was performed to examine migration properties of Wharton's jelly-derived mesenchymal stem cell conditioned medium (WJ-MSC-CM)-treated and untreated normal skin fibroblasts. The treated group showed significantly enhanced migration rates and coapted wound borders faster than the control group. *P ≤ 0.05.

In our culture conditions, WJ-MSC-CM-treated normal skin fibroblasts coapted wound borders faster than normal skin fibroblasts from the control group (that is, those treated with WJ-MSC nonconditioned medium), suggesting that WJ-MSC-CM enhanced fibroblast migration. This observation is important, because cell migration is an essential step during wound healing. This fact highlights that healing promotion is partly due to faster migration, and consequently acceleration of wound closure, in human normal skin fibroblasts (Figure 3A,B).

### Wharton's jelly-derived MSC conditioned medium promotes wound healing and repair in a mouse model

**Figure 4:**
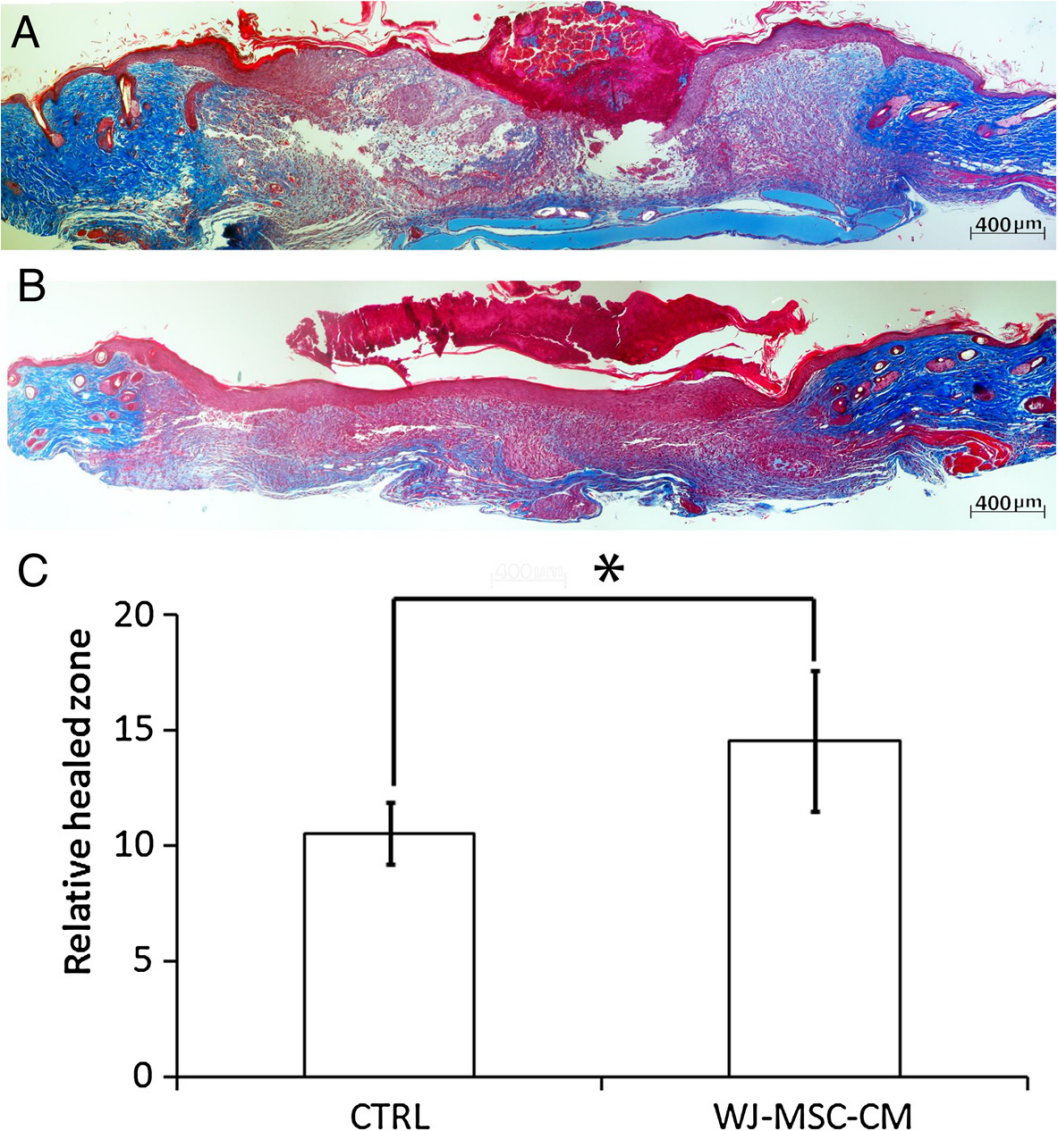
**Wharton's jelly-derived mesenchymal stem cell conditioned medium enhances wound healing in an *in vivo *mouse model.** A murine wound healing model was used, and animals were wounded and treated as described previously. Images corresponded to histological sections of wounds and the satellite donut area (10×) from BALB-c mice, after 1 week of full-thickness excisional skin wounding and reconstruction with Wharton's jelly-derived mesenchymal stem cell conditioned medium (WJ-MSC-CM) and vehicle (Matrigel; BD Biosciences, San Jose, CA, USA) **(B)**, or vehicle alone **(A)**. Photomicrographs were taken after Masson’s Trichrome staining. BALB-c mice WJ-MSC-CM-treated wounds showed enhanced wound healing rates compared to the control mice (*P* ≤ 0.05) **(C)**. *P ≤ 0.05. Error bars represent the 95% confidence interval.

We next examined whether the *in vitro* wound healing promoting observed effects with WJ-MSC-CM might be translated into an *in vivo* wound healing model. BALB-c mice with WJ-MSC-CM-treated wounds showed enhanced wound healing rates compared with the control mice (*P* ≤ 0.05) (Figure 4A,B, quantified in 4C). Increased and complete re-epithelialization, higher cellularity in newly formed granulation tissue, and less random and more organized extracellular matrix were observed in the WJ-MSC-CM-treated wounds, suggesting that WJ-MSC-CM promoted wound healing and repair *in vivo* in mice.

**Figure 5:**
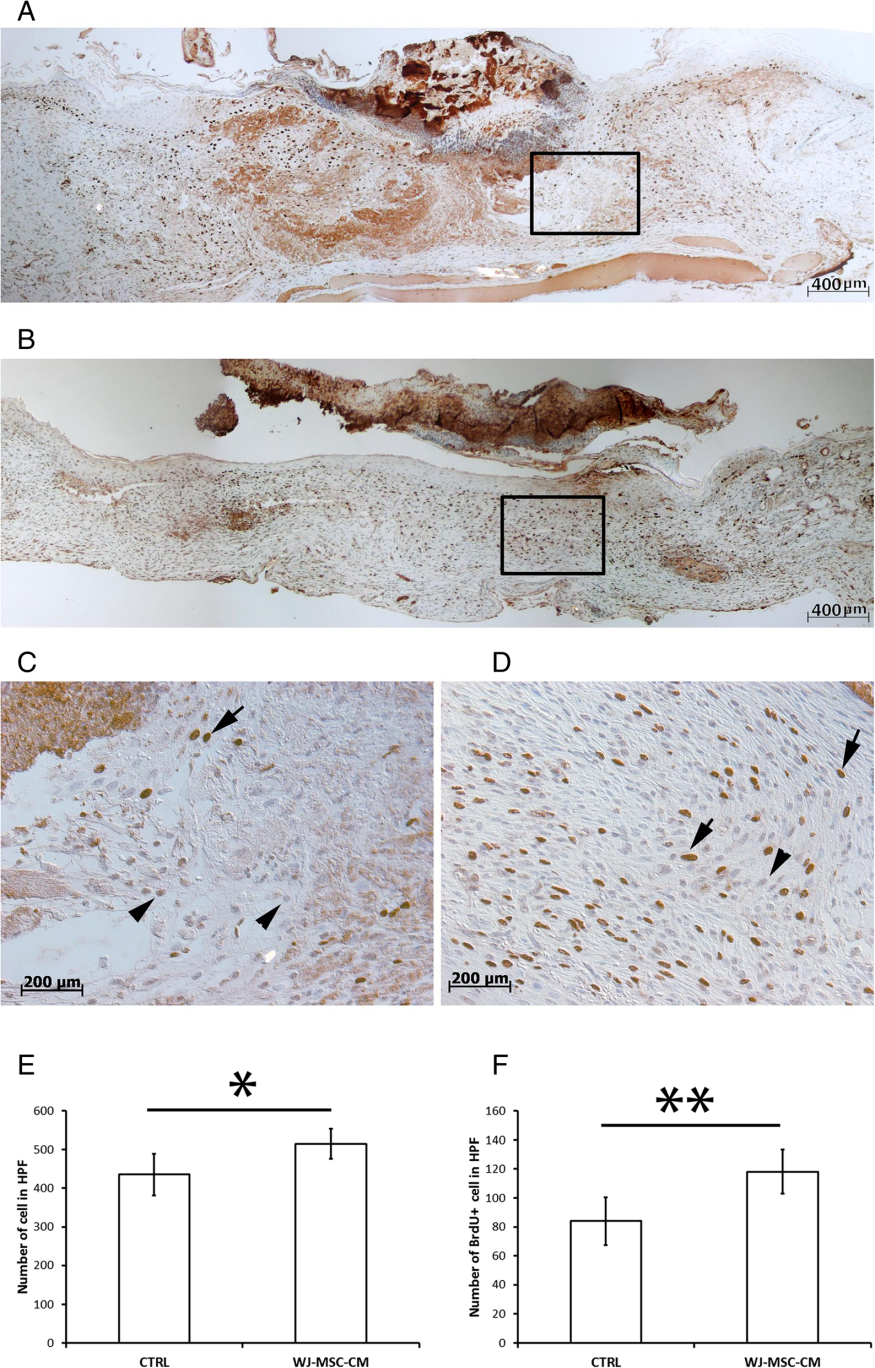
**Wharton's jelly-derived mesenchymal stem cell conditioned medium promotes cell proliferation in an *in vivo *mouse wound healing model.** A BALB-c mouse wound healing model was used and animals were wounded and treated as described previously. Animals received one dose of bromodeoxyuridine (BrdU) intraperitoneally 24 hours before harvesting of wounds. Four animals were included in each group, and four wounds were performed per animal (total of 16 wounds in each group). Cutaneous tissue specimens were stained for BrdU in both groups, control **(A)** and treatment **(B)**. Enhanced magnification (40×) of the above microscopic images were included for nonconditioned medium-treated **(C)** and Wharton's jelly-derived mesenchymal stem cell conditioned medium (WJ-MSC-CM)-treated normal skin fibroblasts **(D)** to examine in further detail the increase in cell number or stained nuclei (black arrows, BrdU-positive cells) in the WJ-MSC-CM-treated wounds, compared with controls. This denoted that WJ-MSC-CM stimulated cell proliferation *in vivo***(E, F)**. Together, these results suggest that WJ-MSC promoted wound healing and repair by one-way paracrine signaling in an *in vivo* preclinical model. *P ≤ 0.05, **P ≤ 0.01. Error bars represent the 95% confidence interval. Arrows, BrdU-positive nuclei; arrowheads, BrdU-negative nuclei. HPF, high-power field.

To delineate the pro-proliferative effect of WJ-MSC-CM *in vivo*, one dose of BrdU was injected intraperitoneally. Both a higher number of cells and a higher amount of proliferative cells were found in the WJ-MSC-CM-treated wounds (*P* ≤ 0.05 and *P* ≤ 0.01, respectively) (Figure 5E,F). A microscopic wound cross-section showed a higher number of positive proliferating nuclei (black arrows, BrdU-positive cells) (*P* ≤ 0.01) in the WJ-MSC-CM-treated wounds (Figure 5D) compared with the control wounds (Figure 5C), as well as general increased cellularity, matrix remodeling and overall wound repair. Higher magnification images corresponding to the aforementioned detailed micrographs of both control and treated wounds were shown in Figure 5A and 5B, respectively (Additional file 3 supports and complements Figure 5).

## Discussion

The results of this study suggest that WJ-MSCs enhance wound healing and normal wound repair by paracrine signaling mechanisms. Under our culture conditions, human WJ-MSC-CM upregulated the gene expression of wound healing factors in human normal skin fibroblasts, and promoted fibroblast proliferation and migration to coapt wound borders *in vitro*. Accordingly, WJ-MSC-CM accelerated the re-epithelialization rate and promoted wound repair *in vivo* in a full-thickness excisional mouse wound healing model.

Cutaneous wound repair is a complex orchestrated process that is activated upon injury and includes the multicellular overlapping and coordinated phases of inflammation, angiogenesis and formation of granulation tissue, re-epithelialization, and fibroproliferation or matrix formation and remodeling [14,15]. Decreased proinflammatory cytokines, compromised neovascularization and/or impairment in leukocyte recruitment might disturb and delay wound healing [16]. Despite the current use and availability of a wide array of wound dressings, ointments and devices, wound healing still remains a clinical challenge, especially in older patients, diabetic patients, heavy smokers or burned patients [17]–[21]. There is therefore a need for new strategies to promote or at least coadjuvantly help in wound healing and repair.

Skin MSCs have been reported to populate the normal skin niche, remain quiescent and become active after injury, aiding in wound closure [14,15]. MSC paracrine signaling has been suggested to be the main underlying mechanism for MSCs enhanced wound repair effects [2,22,23]. BM-MSCs have been reported to promote wound healing, but their isolation requires an invasive and artificial method [24,25]. Harvesting adipose-derived stem cells also requires a surgical procedure. Subsequently, we focused on a more advantageous MSC source, the umbilical cord-derived Wharton’s jelly [5,25,26]. The harvest of WJ-MSCs is not painful or invasive, as the cells are isolated naturally with no extra surgery and are dissected from discarded umbilical cords after birth. Indeed, caprine WJ-MSCs have already been shown to promote wound repair with minimal scarring [6], but to our knowledge there are no reports of human WJ-MSC treatment on human skin wounds. WJ-MSCs represent a very efficient stem cell source with reported immunoprivileged, anticancer and antifibrotic characteristics in animal models [25]–[28]. Due to their reported immunoprivileged properties and universal and ever-lasting availability [5], WJ-MSC allotransplantion as an off-the-shelf therapy may represent an appropriate treatment strategy in the already compromised patients who suffer of recalcitrant cutaneous wounds [14].

This study therefore aimed to examine the effects of human WJ-MSC paracrine signaling on human normal skin fibroblasts. In our culture conditions, human WJ-MSCs enhanced the expression of some wound healing promoting genes, including re-epithelialization, neovascularization and fibroproliferation inducing genes, in human normal skin fibroblasts. Besides altering skin fibroblast gene expression, MSC signaling has also been shown to positively regulate cell survival, proliferation and migration [23,29]. Under our culture conditions, although apoptotic changes were not found in skin fibroblasts treated with WJ-MSC-CM, significantly enhanced proliferation and migration were observed. This is important, as cellular dynamics and cell migration constitute an essential step during cutaneous healing. WJ-MSC-CM accelerated wound closure both *in vitro* and in an *in vivo* mouse wound healing model, suggesting that human WJ-MSC-CM may promote wound repair.

Other researchers have also reported enhanced wound healing rates in mouse models with other human MSC sources, such as BM-MSCs [23,30] and umbilical cord blood-derived MSCs [31]. Blood of the umbilical cord has been reported to yield fewer MSCs than the cord itself [31], however, and Wharton’s jelly may therefore emerge as a more appropriate stem cell source. To the best of our knowledge, no published studies regarding the use of human WJ-MSCs in human wounds have been reported, so this work is novel, and could prompt us to test whether the same results would be encountered first in a more appropriate but more costly experimental model, such as the red duroc pig. Secondly, if the results appeared to be promising, the next step would be to translate them into phase I or phase II clinical trials for further evaluation and development. Indeed, pilot clinical studies have so far indicated that MSCs in general are safe *in vivo*, and they currently represent the most widely used stem cells in the clinical setting [26]. Accordingly, in the particular case of WJ-MSCs, 15 diabetic patients received systemically WJ-MSCs with no documented relevant safety concerns [32]. Furthermore, many MSC clinical applications to manage intestinal fistulae, acute artery ischemic disease in diabetic patients, and periodontal defects have been reported, among others [26]. Anecdotally, one case report about autologous BM-MSCs used to help to heal a scapular recalcitrant wound in a Russian burned patient has also been published [33], but no reports regarding the use of WJ-MSCs have yet been reported. Although WJ-MSCs secrete a more angiogenic secretome in comparison with BM-MSCs [34], the isolation of WJ-MSCs is more efficient, and WJ-MSCs have a higher capacity of proliferation and are less senescent than adipose-derived stem cells [35], proper studies comparing the wound healing abilities of different MSC sources are still lacking in the literature. On the other hand, the direct effects of human WJ-MSCs on wound healing, regeneration and repair still remain unknown. A new preclinical and clinical research arena investigating the potential of WJ-MSCs in wounds and wound healing has just been born and may hold promise for future medical therapies.

## Conclusions

Human WJ-MSCs promoted wound healing by paracrine signaling in our culture conditions *in vitro*, and in an *in vivo* preclinical animal model. If the reported immunoprivileged character and safety of WJ-MSCs observed in experimental and first clinical models is further scientifically proven, WJ-MSCs might represent a feasible, universal and off-the-shelf technology to enhance normal wound healing to improve patient survival and quality of life.

## Abbreviations

BM-MSC: bone marrow-derived mesenchymal stem cell; BrdU: bromodeoxyuridine; CM: conditioned medium; DAPI: 4′,6-diamidino-2-phenylindole; DMEM: Dulbecco’s modified Eagle’s medium; FBS: fetal bovine serum; IL: interleukin; MSC: mesenchymal stem cell; PBS: phosphate-buffered saline; PCR: polymerase chain reaction; TGF-β: transforming growth factor beta; VEGF: vascular endothelial growth factor; WJ-MSC: Wharton’s jelly-derived mesenchymal stem cell.

## Competing interests

The authors declare that they have no competing interests or any potential conflict of interest in any of the techniques or instruments mentioned in this manuscript.

## Authors’ contributions

AIA participated in the conception and design, administrative support, collection and/or assembly of data, data analysis and interpretation, manuscript writing and final approval of the manuscript. SA-N contributed to the conception and design, administrative support, collection and/or assembly of data, data analysis and interpretation, manuscript writing and final approval of the manuscript. PHB participated in the conception and design, administrative support, collection and/or assembly of data, data analysis and interpretation, manuscript writing and final approval of the manuscript. MA-S contributed to administrative support, collection and/or assembly of data and final approval of the manuscript. CB provided administrative support and helped in collection and/or assembly of data and final approval of the manuscript. EH carried out provision of study material or patients, administrative support, helped in collection and/or assembly or data and contributed to final approval of the manuscript. CHT provided financial and administrative support and study material, helped in collection of data and approved final version of the manuscript. MGJ carried out conception and design, financial support, administrative support, provision of study material or patient collection and/or assembly of data, data analysis and interpretation, manuscript writing and final approval of the manuscript. All authors read and approved the final manuscript.

## Supplementary Material

Additional file 1: Figure S1 Showing flow-cytometry markers and mesenchymal differentiation of human WJ-MSCs. Flow cytometry analysis of established human WJ-MSCs (successfully grown on plastic plates) showing markers used to characterize MSCs (a,b,c,d). Cells were able to differentiate into osteocytes (e), chondrocytes (f) and adipocytes (g). Images shown after alizarin red (e), safranin O (f) and oil red (g) staining, respectively. Click here for file

Additional file 2: Table S1 Showing primer sequences for real-time PCR. List of SYBR® Green gene primer sequences used in real-time PCR for the present work. *TGF-β, transforming growth factor-β; CTGF, connective tissue growth factor; PAI-I, plasminogen activator inhibitor-I; HIF-1-α, hypoxia inducible factor-1-α; VEGF, vascular endothelial growth factor; FGF-2, fibroblast growth factor-2. Click here for file

Additional file 3: Figure S2 Showing WJ-MSC-CM promoted cell proliferation in an *in vivo* wound healing model in BALB-c mice. (A) BrdU staining in the control group. (B) BrdU staining in the treatment group. (C), (D) Higher magnification (40×) of the above microscopic images were included for nonconditioned medium-treated (C is magnification of the marked area in A) and WJ-MSC-CM-treated normal skin fibroblasts (D is magnification of the marked area in B) to examine in further detail the increase in cell number or stained nuclei in the WJ-MSC-CM-treated wounds, compared with controls. Brown nuclei correspond to cells that incorporated BrdU into their DNA. (Supplemental figure in support of Figure 5). Click here for file
