# Supplementary material for: Human Wharton’s jelly mesenchymal stem cells promote skin wound healing through paracrine signaling
Source: Stem Cell Res Ther. 2014 Feb 24;5(1):28. doi: 10.1186/scrt417 (PMC4055091; doi:10.1186/scrt417)
Supplement: Additional file 1: Figure S1 — Showing flow-cytometry markers and mesenchymal differentiation of human WJ-MSCs. Flow cytometry analysis of established human WJ-MSCs (successfully grown on plastic plates) showing markers used to characterize MSCs (a,b,c,d). Cells were able to differentiate into osteocytes (e), chondrocytes (f) and adipocytes (g). Images shown after alizarin red (e), safranin O (f) and oil red (g) staining, respectively. [file scrt417-S1.pptx]

## Slide 1
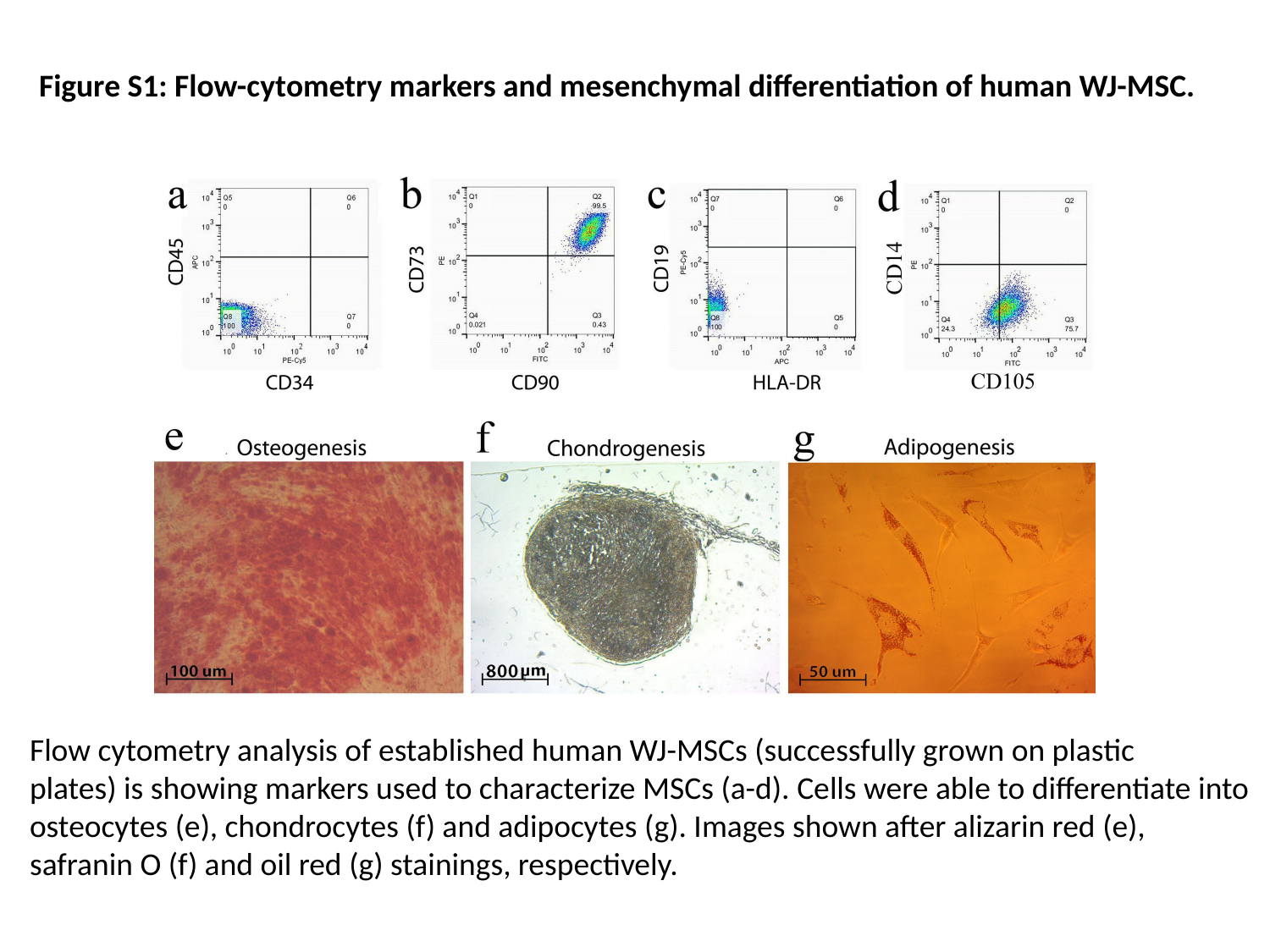

Figure S1: Flow-cytometry markers and mesenchymal differentiation of human WJ-MSC.
 Flow cytometry analysis of established human WJ-MSCs (successfully grown on plastic
 plates) is showing markers used to characterize MSCs (a-d). Cells were able to differentiate into
 osteocytes (e), chondrocytes (f) and adipocytes (g). Images shown after alizarin red (e),
 safranin O (f) and oil red (g) stainings, respectively.
