# Supplementary material for: Human Wharton’s jelly mesenchymal stem cells promote skin wound healing through paracrine signaling
Source: Stem Cell Res Ther. 2014 Feb 24;5(1):28. doi: 10.1186/scrt417 (PMC4055091; doi:10.1186/scrt417)
Supplement: Additional file 2: Table S1 — Showing primer sequences for real-time PCR. List of SYBR® Green gene primer sequences used in real-time PCR for the present work. *TGF-β, transforming growth factor-β; CTGF, connective tissue growth factor; PAI-I, plasminogen activator inhibitor-I; HIF-1-α, hypoxia inducible factor-1-α; VEGF, vascular endothelial growth factor; FGF-2, fibroblast growth factor-2. [file scrt417-S2.pdf]

Table S1: Primer sequences for RT-PCR.

Gene	Forward primer sequence	Reverse sequence primer
TGF-β1	GGCTTTCGCCTTAGCGCCCA	CTCGGCGGCCGGTAGTGAAC
TGF-β2	CTTTGGATGCGGCCTATTGCT	AGCTGTTCAATCTTGGGTGTTT
TGF-β3	GCTGAGACCCCACGTGCGAC	GTGTTTCCCGAGGAGCGGGC
CTGF	TGCCCGGGAAATGCTGCGAG	CAGTCGGTAAGCCGCGAGGG
PAI-1	TGGCACGGTGGCCTCCTCAT	TCCTGTGGGGTTGTGCCGGA
HIF-1-α	GATCACCCTCTTCGTCGCTT	AAGGAAAGGCAAGTCCAGAGG
VEGF	ACGAAAGCGCAAGAAATCCC	CTCCAGGGCATTAGACAGCA
FGF-2	CTGGCTATGAAGGAAGATGGA	TGCCCAGTTCGTTTCAGTG
Decorin	CGCCTCATCTGAGGGAGCTT	TACTGGACCGGGTTGCTGAA
Collagen type I	GGCCAAGACGAAGACATCCCACCAA	TGCCGTTGTCGCAGACGCAGAT
Collagen type III	GAGGTGGTGCAGGTGAGCCTGGTAA	GATCCATCCTTGCCATCTTCGCCTT
18S	GTAACCCGTTGAACCCCATT	CCATCCAATCGGTAGTAGCG

Figure legend:

List of SYBR® Green gene primer sequences used in real time PCR for present work.

* Abbreviations: *TGF-β: Transforming Growth Factor- β; CTGF: Connective Tissue Growth Factor; PAI-I: Plasminogen Activator Inhibitor-I; HIF-1-α: Hypoxia Inducible Factor-1-α; VEGF: Vascular Endothelial Growth Factor; FGF-2: Fibroblast Growth Factor-2.*
