# Supplementary material for: Human Wharton’s jelly mesenchymal stem cells promote skin wound healing through paracrine signaling
Source: Stem Cell Res Ther. 2014 Feb 24;5(1):28. doi: 10.1186/scrt417 (PMC4055091; doi:10.1186/scrt417)
Supplement: Additional file 3: Figure S2 — Showing WJ-MSC-CM promoted cell proliferation in an in vivo wound healing model in BALB-c mice. (A) BrdU staining in the control group. (B) BrdU staining in the treatment group. (C), (D) Higher magnification (40×) of the above microscopic images were included for nonconditioned medium-treated (C is magnification of the marked area in A) and WJ-MSC-CM-treated normal skin fibroblasts (D is magnification of the marked area in B) to examine in further detail the increase in cell number or stained nuclei in the WJ-MSC-CM-treated wounds, compared with controls. Brown nuclei correspond to cells that incorporated BrdU into their DNA. (Supplemental figure in support of Figure 5). [file scrt417-S3.pptx]

## Slide 1
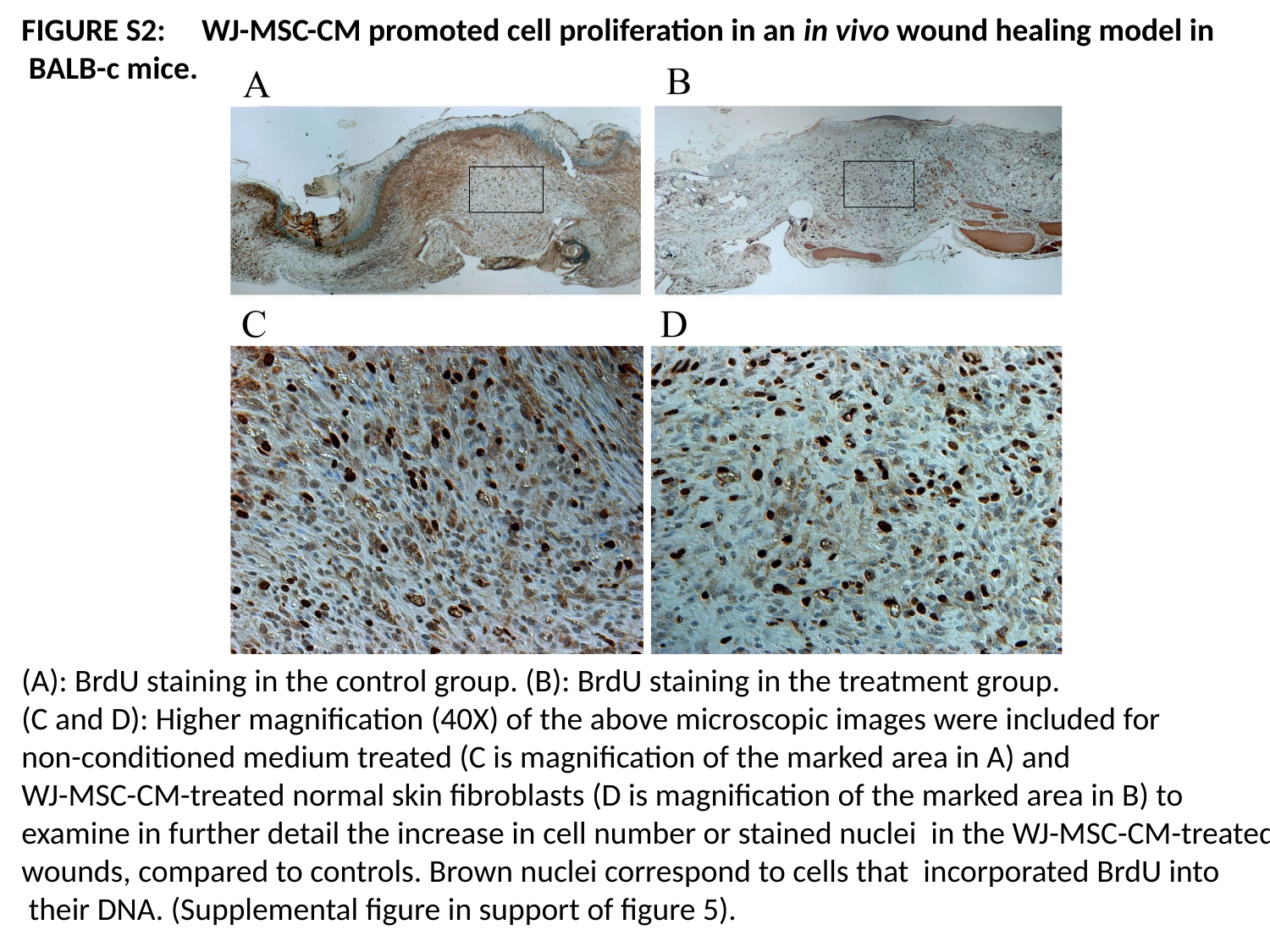

FIGURE S2: WJ-MSC-CM promoted cell proliferation in an in vivo wound healing model in
 BALB-c mice.
(A): BrdU staining in the control group. (B): BrdU staining in the treatment group.
(C and D): Higher magnification (40X) of the above microscopic images were included for
non-conditioned medium treated (C is magnification of the marked area in A) and
WJ-MSC-CM-treated normal skin fibroblasts (D is magnification of the marked area in B) to
examine in further detail the increase in cell number or stained nuclei  in the WJ-MSC-CM-treated
wounds, compared to controls. Brown nuclei correspond to cells that incorporated BrdU into
 their DNA. (Supplemental figure in support of figure 5).
